# The Association of Therapeutic Alliance With Long-Term Outcome in a Guided Internet Intervention for Depression: Secondary Analysis From a Randomized Control Trial

**DOI:** 10.2196/15824

**Published:** 2020-03-24

**Authors:** Juan Martín Gómez Penedo, Anna Margarete Babl, Martin grosse Holtforth, Fritz Hohagen, Tobias Krieger, Wolfgang Lutz, Björn Meyer, Steffen Moritz, Jan Philipp Klein, Thomas Berger

**Affiliations:** 1 Department of Clinical Psychology and Psychotherapy University of Bern Bern Switzerland; 2 Department of Psychosomatic Medicine, Inselspital University of Bern Bern Switzerland; 3 Department of Psychiatry and Psychotherapy Lübeck University Lübeck Germany; 4 Department of Clinical Psychology and Psychotherapy University of Trier Trier Germany; 5 Department of Psychology City, University of London London United Kingdom; 6 Department of Psychiatry and Psychotherapy University Medical Center Hamburg-Eppendorf Hamburg Germany

**Keywords:** internet interventions, guidance, alliance, Working Alliance Inventory for Guided Internet Intervention, only interventions, tasks and goals, bond

## Abstract

**Background:**

Therapeutic alliance has been well established as a robust predictor of face-to-face psychotherapy outcomes. Although initial evidence positioned alliance as a relevant predictor of internet intervention success, some conceptual and methodological concerns were raised regarding the methods and instruments used to measure the alliance in internet interventions and its association with outcomes.

**Objective:**

The aim of this study was to explore the alliance-outcome association in a guided internet intervention using a measure of alliance especially developed for and adapted to guided internet interventions, showing evidence of good psychometric properties.

**Methods:**

A sample of 223 adult participants with moderate depression received an internet intervention (ie, Deprexis) and email support. They completed the Working Alliance Inventory for Guided Internet Intervention (WAI-I) and a measure of treatment satisfaction at treatment termination and measures of depression severity and well-being at termination and 3- and 9-month follow-ups. For data analysis, we used two-level hierarchical linear modeling that included two subscales of the WAI-I (ie, *tasks and goals* agreement with the program and *bond* with the supporting therapist) as predictors of the estimated values of the outcome variables at the end of follow-up and their rate of change during the follow-up period. The same models were also used controlling for the effect of patient satisfaction with treatment.

**Results:**

We found significant effects of the *tasks and goals* subscale of the WAI-I on the estimated values of residual depressive symptoms (γ_02_=−1.74, standard error [SE]=0.40, 95% CI −2.52 to −0.96, t_206_=−4.37, *P*<.001) and patient well-being (γ_02_=3.10, SE=1.14, 95% CI 0.87-5.33, t_198_=2.72, *P*=.007) at the end of follow-up. A greater score in this subscale was related to lower levels of residual depressive symptoms and a higher level of well-being. However, there were no significant effects of the *tasks and goals* subscale on the rate of change in these variables during follow-up (depressive symptoms, *P*=.48; patient well-being, *P*=.26). The effects of the *bond* subscale were also nonsignificant when predicting the estimated values of depressive symptoms and well-being at the end of follow-up and the rate of change during that period (depressive symptoms, *P*=.08; patient well-being, *P*=.68).

**Conclusions:**

The results of this study point out the importance of attuning internet interventions to patients’ expectations and preferences in order to enhance their agreement with the tasks and goals of the treatment. Thus, the results support the notion that responsiveness to a patient’s individual needs is crucial also in internet interventions. Nevertheless, these findings need to be replicated to establish if they can be generalized to different diagnostic groups, internet interventions, and supporting formats.

## Introduction

Several meta-analyses positioned therapeutic alliance as a robust predictor of outcomes in face-to-face psychotherapy [1-3]. However, alliance effects do not seem to be limited to the field of traditional psychotherapy. Alliance also predicted outcomes in other health-related interventions, such as pharmacotherapy [4,5]. The increasing development of internet interventions and the evidence for their efficacy and effectiveness in treating diverse mental disorders [6-8] raised the question of what role therapeutic alliance might play in such treatments, especially in those providing guidance from trained supporters (guided internet interventions) [9]. In the last years, several studies addressed this question scientifically. Although some authors found that the alliance could be less important in internet interventions than in traditional face-to-face therapies [10], a recent meta-analysis reported similar effect sizes (*r*=0.275) for the alliance-outcome relationship in online interventions as in traditional face-to-face therapies [1]. Among the 18 studies included in that meta-analysis [1], 15 analyzed the alliance specifically in guided internet interventions. To measure the alliance, most of the studies used the same instruments usually administered in face-to-face psychotherapy (ie, the Working Alliance Inventory [WAI]) [11] with very slight modifications (eg, talking about a treatment instead of therapy) and focused on the effects of the relationship between the patient and the supporting therapist [10,12-21]. This approach of measuring alliance follows its classical conceptualization in psychotherapy research as tripartite, consisting of (1) the patient-therapist emotional bond, (2) patient agreement with the tasks of therapy, and (3) patient agreement with the therapeutic goals that the patient and therapist seek in treatment [22,23]. However, it was not considered that in these trials, treatment tasks and goals were not set in collaboration with the supporting therapist but were proposed by the online program, which might be a limiting factor. Some studies did this the other way around; they used adapted instruments to measure the alliance between the patient and the online intervention only [18,24-26].

Regardless of whether the measuring instruments focused on the supporting therapist or the program, all the abovementioned studies have one thing in common. They lack an exploration of the psychometric properties (ie, validity and reliability) of the measuring instruments used in the specific context of guided online interventions. Only Kiluk et al [24] presented an exploratory analysis of the adapted version of the WAI (ie, WAI-Tech) with some evidence of internal consistency (Cronbach alpha) and external validity (ie, significant correlations with the original WAI and with patient treatment satisfaction). However, the small sample size of that study (n=34) limited the interpretability of the findings and prevented the provision of further evidence for the psychometric properties of the scale (eg, construct validity). Thus, beside conceptual issues identified in most previous alliance-outcome studies of guided internet interventions, concerns might be raised regarding the validity and reliability of the instruments used to measure the alliance construct.

In this context, Berger et al [27], as well as Scherer et al [28], presented a compromise between the two previous approaches of analyzing alliance-outcome associations in guided internet interventions (focus on the supporting therapist or the online program). The authors took the original version of the WAI and adapted it to guided internet interventions, exploring the bond with the supporting therapist but the tasks and goals with the online program. With this version of the instrument, they captured the most relevant aspects of the alliance, considering both the importance of the relationship with the supporting therapist and patient attunement with the online program. Recently, Gómez Penedo et al [29] systematized the efforts by Berger et al [27] and Scherer et al [28], presenting the Working Alliance Inventory for Guided Internet Interventions (WAI-I) and exploring the psychometric properties of the scale. The findings provide evidence for adequate internal consistency, external validity, and construct validity (based on a confirmatory factor analysis) of the WAI-I.

Given the cumulative evidence showing that residual depressive symptoms are some of the main predictors of relapse [30-32], in this study, we will analyze how the alliance during treatment is associated with long-term outcomes (ie, 9-month follow-up) after a guided internet intervention for patients with moderate depressive symptoms. We will focus on both analyzing the effects of the alliance on the changes produced during the follow-up period (ie, deterioration or further improvement) and evaluating the residual depressive symptoms at the 9-month follow-up. Furthermore, we will analyze the same effects on the well-being of patients. Beside responding to a general call for further studies clarifying the role of alliance in online interventions [1,9], especially with adapted and psychometrically sound instruments [29], the aim of this study was to explore the alliance-outcome association using the WAI-I, a measure of alliance especially developed for and adapted to guided internet interventions.

## Methods

### Participants

For this study, we drew on a dataset from the EVIDENT study [33,34], a large multicenter randomized controlled trial analyzing the effects of the internet intervention Deprexis [35]. In that trial, 509 patients were assigned to the online intervention. Of these 509 participants, 317 presented with moderate depressive symptoms (ie, score between 10 and 14 in the Patient Health Questionnaire [PHQ]) [36] and therefore additionally received weekly email support from trained clinicians. In our study, we analyzed a sample of 223 patients who received both Deprexis and email support, and completed the WAI-I at treatment termination. Patients in this study were aged between 18 and 65 years (mean 44.48 [SD 10.68] years) and were German speaking. Furthermore, most participants were female (157/223, 70.4%), had high school education (107/223, 48.0%), and were in a romantic relationship at the beginning of the treatment (151/223, 67.7%). The exclusion criteria were a lifetime diagnosis of a bipolar disorder or schizophrenia and acute suicidality established by a telephone diagnostic interview.

### Internet Intervention

Deprexis is an internet intervention that demonstrated effectiveness when treating depression, showing medium effect sizes at posttreatment [37]. Deprexis has 10 modules (in addition to one introductory and one summary module) developed consistent with cognitive-behavioral treatment manuals. Within the modules, the program provides simulated dialogues, explaining different techniques and key concepts and delivering examples, and illustrations for easy understanding. Participants were requested to complete different exercises within the program and provide feedback in order to attune the interventions to each participant. Although the modules were presented sequentially, patients could repeat them as often as they wanted. In this sample, the participants spent a mean of 520 (SD 314) minutes in the program and completed a mean of 9.74 (SD 4.51) modules.

All the participants in this sample received standardized email support that consisted of weekly feedback regarding their activity on Deprexis during the last week. The main goal of this support was to enhance participants’ motivation and engagement with the internet intervention. The support was implemented via a secured email system included in the internet intervention and was delivered by master’s students in clinical psychology and psychotherapy, psychotherapists in training, and licensed psychotherapists who received an intensive 4-hour training in the program and feedback strategies, using example cases. The instructions provided to the supporters were in line with those used in a similar previous trial [38]. An expert on internet interventions supervised their tasks by periodically revising the messages from the supporters and providing feedback to them. The study participants were able to contact the supporters directly or respond to their messages.

The sample of this study received a mean of 12.11 (SD 3.23) messages from the supporting clinicians and read a mean of 9.65 (SD 4.88) of those messages. Additionally, the patients sent a mean of 1.99 (SD 2.84) messages to the supporting clinicians (54.7% of the sample sent at least one message). Further details on the internet intervention Deprexis are presented in articles by Meyer et al [35] and Klein et al [33,34].

### Measures

#### Working Alliance Inventory for Guided Internet Interventions

The WAI-I is an instrument derived from the Working Alliance Inventory-Short revised [39], and it was specifically adapted to guided internet interventions [27,29]. The WAI-I is a 12-item self-reported measure rated on a 5-point Likert scale ranging from 1 (never) to 5 (always). The instrument has two dimensions. One dimension explores the emotional bond between the patient and the supporting therapist, with items like “I feel that the psychologist who supports me in the online program appreciates me.” The second dimension analyzes patient agreement with the tasks and goals of the internet intervention, with items like “I believe the way the online program is working with my problem is correct” and “The goals of the online program are important goals for me.” The instrument showed evidence of adequate internal consistency, external validity, and construct validity for a two-factor solution (based on a confirmatory factor analysis) [29]. In this sample, the Cronbach alpha of the *bond* subscale was .89, whereas that of the *tasks and goals* subscale was .93.

#### Patient Satisfaction Questionnaire (Zurich Satisfaction Questionnaire-8)

To measure patient satisfaction with treatment, we used the Zurich Satisfaction Questionnaire-8 (ZUF-8) questionnaire [40] adapted for internet interventions [33]. This instrument is a self-reported measure of eight items rated on a 4-point Likert scale ranging from 1 (low satisfaction) to 4 (high satisfaction). The ZUF-8 adapted to the German culture showed good psychometric properties with evidence of internal consistency, concurrent validity, and construct validity [40]. In the sample of this study, the ZUF-8 showed a Cronbach alpha of .92.

#### Patient Health Questionnaire-9

The PHQ-9 is a widely used outcome measure for the treatment of depression [41]. It has nine self-reported items representing the nine Diagnostic and Statistical Manual of Mental Disorders-version IV criteria of depression that are completed on a 4-point Likert scale from 0 (not at all) to 3 (nearly every day). Previous studies showed that it is a reliable and valid instrument to measure depression severity [41]. In this study, the PHQ-9 presented good internal consistency during follow-up, with a Cronbach alpha of .82.

#### Short-Form Health Survey-12

The Short-Form Health Survey-12 (SF-12) is an instrument for measuring health-related quality of life [42]. For this study, we used the mental health subscale (SF-P) of this measure, which consisted of six items, with higher scores representing a higher quality of life in terms of mental health. The items from SF-12 have a Likert scale that varies from 3 to 6 response categories, depending on the item. The individual item responses are then transformed into a 0 to 100 scale and then aggregated into different dimensions or subscales, with higher scores representing greater health well-being. Thereafter, the aggregated scores are standardized according to a normative population by computing *t*-scores that have a mean of 50 and an SD of 10 [34]. The SF-12 showed evidence of reliability and validity [42]. In this sample, the SF-P presented good internal consistency, with a Cronbach alpha of .84.

### Procedure

Patients completed the alliance measure (WAI-I) only once at posttreatment. We decided to measure alliance only at the end of therapy, as some studies showed that patients might have difficulties to complete it early in treatment, because of the limited interaction with the supporter during the intervention [16]. At treatment termination, patients also completed the ZUF-8 as a general measure of patient satisfaction. Furthermore, they completed the PHQ-9 and SF-12 at posttreatment and at 3- and 9-month follow-ups. The Ethics Committee of the German Psychological Association approved the procedure of the study (Deutsche Gesellschaft für Psychologie, reference number SM 04_2012). All patients completed an electronic informed consent form before baseline assessments.

### Analytic Strategy

For the analyses in this study, we used hierarchical linear models (HLMs) to deal with the dependency of the observations owing to the nestedness of the data [43]. Considering that repeated measures during follow-up were nested within patients, we ran two-level HLMs, accounting for within-patient and between-patient variabilities. These models accommodate missing data, allowing to retain in the analyses all patients with at least one measurement point, which mimics an intent-to-treat approach.

We first ran two-level fully unconditional models with PHQ-9 and SF-12 values during follow-up as the dependent variables. In the next step, we ran an unconditional time-as-only predictor model, with time as a level 1 predictor centered at the 9-month follow-up and representing the evolution during the follow-up period (posttreatment=−1; end of follow-up=0). Thereafter, we ran a conditional model that included WAI-I scores in the *bond* and *tasks* and *goals* subscales as separate level 2 predictors of the intercept (ie, estimated score of the outcome variables at the 9-month follow-up) and the linear slope of time (ie, evolution of the outcome variables during follow-up). Finally, we ran the exact same models but included either (1) patient satisfaction with treatment or (2) participant-supporter interaction indicators (ie, number of messages sent by the participant, number of messages sent by the supporter, and number of messages read by the participant) as a level 2 predictor to control for its effect.

## Results

### Sample Details

To characterize the sample, we calculated the mean and SD of each of the targeted variables at posttreatment. We have presented these descriptive statistics in Table 1.

Furthermore, in Multimedia Appendix 1, we present the correlations among these variables at posttreatment. Beside these correlations among the targeted variables of the study, we found significant correlations between the use of the program in minutes and both the *bond* subscale (*r*=0.18, *P*=.01) and the *tasks and goals* subscale (*r*=0.16, *P*=.02) of the WAI-I. In addition, the number of modules performed by the participants was significantly related to the *bond* subscale (*r*=0.22, *P*=.001) and the *tasks and goals* subscale (*r*=0.15, *P*=.02).

**Table 1:** Sample details at posttreatment for the variables in the study.

Measures		Value, mean (SD)
**WAI-I^a^**		
	T&G^b^ subscale	3.17 (0.91)
	Bond subscale	3.56 (1.15)
**ZUF-8^c^**		
	Total scale	3.13 (0.59)
**PHQ-9^d^**		
	Total scale	7.44 (4.32)
**SF-12^e^**		
	SF-P^f^	38.22 (11.75)

^a^WAI-I: Working Alliance Inventory for Internet Interventions.

^b^T&G: task and goal subscale.

^c^ZUF-8: Zurich Satisfaction Questionnaire-8.

^d^PHQ-9: Patient Health Questionnaire-9.

^e^SF-12: Short-Form Health Survey-12.

^f^SF-P: mental health well-being subscale of the Short-Form Health Survey-12.

### Fully Unconditional Model

We present the results of all conducted models in Table 2. The fully unconditional model estimated a mean level of 7.14 units in the PHQ-9 during follow-up (*γ*_00_=7.14, standard error [SE]=0.25, 95% CI 6.65-7.63, t_219_=64.05, *P*<.001). Furthermore, the model for SF-P estimated a mean level of 39.23 units during follow-up (*γ*_00_=39.23, SE=0.68, 95% CI 37.90-40.56, t_217_=57.65, *P*<.001).

**Table 2:** Results of the unconditional, time-as-only predictor, and conditional main effect hierarchical linear models.

Fixed effects			Estimated score at the end of follow-up (*β*_0j_)		Change during follow-up (*β*_1j_)	
			γ	SE^a^	γ	SE
**PHQ^b^ as outcome**						
	**Fully unconditional**					
		Intercept	7.14^c^	0.25	—^d^	—
	**Time-as-only predictor**					
		Intercept	6.85^c^	0.29	−0.49^e^	0.27
	**Main effects of alliance**					
		Intercept	6.84^c^	0.28	−0.49^e^	0.27
		WAI-I^f^ T&G^g^	−1.74^c^	0.40	0.27	0.39
		WAI-I bond	0.57^e^	0.32	0.54^e^	0.31
**SF-P^h^ as outcome**						
	**Fully unconditional**					
		Intercept	39.23^c^	0.68	—	—
	**Time-as-only predictor**					
		Intercept	40.04^c^	0.80	1.33^e^	0.75
	**Main effects of alliance**					
		Intercept	40.19^c^	0.79	1.39^e^	0.76
		WAI-I T&G	3.10^i^	1.14	−1.26	1.10
		WAI-I bond	−0.74	0.91	−0.36	0.89

^a^SE: standard error.

^b^PHQ: Patient Health Questionnaire.

^c^*P*<.001.

^d^Not applicable.

^e^*P*<.10.

^f^WAI-I: Working Alliance Inventory for Internet Interventions.

^g^T&G: task and goal subscale.

^h^SF-P: mental health well-being subscale of the Short-Form Health Survey-12.

^i^*P*<.01.

### Unconditional Time-as-Only Predictor Model

When predicting PHQ-9 scores, the inclusion of the time variable as a level 1 predictor significantly improved the fit of the fully unconditional model (χ^2^_3_=9.70, *P*=.02). The time-as-only predictor model for PHQ-9 estimated a residual depression symptom score of 6.85 at the 9-month follow-up (*γ*_00_=6.85, SE=0.29, 95% CI 6.28-7.42, t_209_=54.84, *P*<.001). This model also showed that the change in the PHQ-9 score during follow-up approached significance (*γ*_10_=−0.49, SE=0.27, 95% CI −1.02 to 0.04, t_203_=−1.80, *P*=.08). The computation of the CIs for the random effects showed significant random effects for both the estimated residual depressive symptoms at the 9-month follow-up (SD 3.33, 95% CI 2.91-3.98) and the change during that period (SD 1.73, 95% CI 0.74-2.80). The results revealed that the findings of the participants significantly varied around the average estimated residual depressive symptoms at the end of the 9-month follow-up and the average rate of change during follow-up, suggesting the inclusion of level 2 predictors to explain this variance.

For the models predicting SF-P, the inclusion of the time variable as a level 1 predictor did not significantly increase the fit of the fully unconditional model (χ^2^_3_=4.17, *P*=.24). This time-as-only predictor model showed an estimated value of 40.04 for the SF-P at the 9-month follow-up (*γ*_00_=40.04, SE=0.80, 95% CI 38.47-41.61, t_200_=50.19, *P*<.001). Furthermore, the change in the SF-P during follow-up approached significance (*γ*_10_=1.33, SE=0.75, 95% CI −0.14 to 2.80, t_190_=1.77, *P*=.08). The calculation of CIs showed significant random effects for both the estimated well-being at the end of follow-up (SD 8.88, 95% CI 7.35-10.55) and the rate of change during follow-up (SD 3.20, 95% CI 0.09-6.32). Thus, the findings of the participants significantly varied around the average estimated well-being at the end of follow-up and the average change during follow-up, suggesting the inclusion of level 2 predictors to explain this variance.

### Conditional Models: Alliance Main Effects

The conditional model with the PHQ-9 as an outcome variable and the alliance subscales as level 2 predictors significantly improved the fit of the time-as-only predictor model (χ^2^_4_=26.71, *P*<.001). This model showed a significant effect of the *tasks and goals* subscale on the estimated PHQ-9 value at the end of follow-up (*γ*_02_=−1.74, SE=0.40, 95% CI −2.52 to −0.96, t_206_=−4.37, *P*<.001). A 1-unit greater score in the *tasks and goals* subscale was associated with a 1.74 lower score in the PHQ-9 at the end of follow-up. However, there was no significant effect of the *tasks and goals* subscale on PHQ-9 change during follow-up (*γ*_12_=0.27, SE=0.39, 95% CI −0.49 to 1.03, t_197_=0.71, *P*=.48). Additionally, the *bond* subscale did not have a significant effect on the estimated score of the PHQ-9 at the end of follow-up (*γ*_01_=0.57, SE=0.32, 95% CI −0.06 to 1.20, t_204_=1.79, *P*=.07) or the change in the PHQ-9 during follow-up (*γ*_11_=0.54, SE=0.31, 95% CI −0.07 to 1.15, t_197_=1.75, *P*=.08).

Furthermore, the conditional model with the SF-P as the outcome variable and the alliance subscales as level 2 predictors significantly improved the model fit as compared with the time-as-only predictor model (χ^2^_4_=20.59, *P*<.001). This conditional model also showed a significant effect of the *tasks and goals* subscale on the estimated SF-P score at the end of follow-up (*γ*_02_=3.10, SE=1.14, 95% CI 0.87-5.33, t_198_=2.72, *P*=.007). A 1-unit greater *tasks and goals* score at posttreatment was associated with a 3.10-unit higher score in the SF-P at the end of the 9-month follow-up. There was no significant effect of the *tasks and goals* subscale on the development of the SF-P during follow-up (*γ*_12_=−1.26, SE=1.10, 95% CI −3.42 to 0.90, t_188_=−1.14, *P*=.26). Moreover, there was no significant effect of the *bond* subscale on the estimated score of the SF-P at the end of follow-up (*γ*_01_=−0.74, SE=0.91, 95% CI −2.52 to 1.04, t_191_=−0.82, *P*=.41) or the evolution of the SF-P during follow-up (*γ*_11_=−0.36, SE=0.89, 95% CI −2.10 to 1.38, t_191_=0.41, *P*=.68).

### Conditional Models: Alliance Main Effects Controlling for Patient Satisfaction and Patient-Supporter Interaction

The results of the conditional models estimating the alliance effects controlling for either patient satisfaction or patient-supporter interaction are presented in Multimedia Appendix 2. When running the same conditional models to predict PHQ-9 scores presented above on controlling for patient satisfaction with treatment, there was a significant improvement in the conditional model fit (χ^2^_2_=8.61, *P*=.01). This model comparison test suggested the importance of controlling for patient satisfaction when estimating the alliance effects on the PHQ-9. The results of this model showed that there was still a significant effect of the *tasks and goals* subscale on the estimated PHQ-9 value at the end of follow-up (*γ*_02_=−1.78, SE=0.58, 95% CI −2.92 to −0.64, t_203_=−3.09, *P*=.002). The other effects of the alliance were nonsignificant as in the previous models. Furthermore, the effect of patient satisfaction was not significant when predicting the estimated PHQ-9 scores at the 9-month follow-up (*γ*_03_=0.09, SE=0.85, 95% CI −1.58 to 1.76, t_200_=0.11, *P*=.91) but was significant when predicting the change produced in the PHQ-9 during follow-up (*γ*_13_=2.00, SE=0.81, 95% CI 0.41-3.59, t_193_=2.48, *P*=.58). When controlling for the alliance subscale effects, a 1-unit greater score in the ZUF-8 at posttreatment (ie, patient satisfaction with treatment) was associated with a 2-unit increase in the PHQ-9 score during the follow-up period.

However, when running a conditional model exploring alliance effects on the PHQ controlling for patient-supporter interaction indicators (ie, number of messages sent by the participant, number of messages sent by the supporter, and number of messages read by the participant), there was no significant improvement in the model fit (χ^2^_6_=0.56, *P*=.99). This test suggested not to include participant-supporter interactions as covariates in conditional alliance models. Nevertheless, it is worth highlighting that the model controlling for participant-supporter interaction indicators still showed significant effects of the *tasks and goals* subscale on the estimated PHQ-9 value at the end of follow-up (*γ*_02_=−1.97, SE=0.44, 95% CI −2.82 to −1.12, t_141_=−4.49, *P*<.001).

Furthermore, inclusion of patient satisfaction in the conditional models predicting SF-P did not significantly improve the model fit from the conditional models that included only alliance subscales (χ^2^_2_=0.39, *P*=.82). This model comparison test again suggested not to include patient satisfaction when estimating alliance effects on the SF-P during follow-up, keeping as the final models the conditional models introduced in the section presented above (ie, conditional models with alliance-only main effects). As can be seen in Multimedia Appendix 1, the results of the model predicting the SF-P and controlling for patient satisfaction suggested no significant effects of either alliance subscales or patient satisfaction.

Additionally, the models controlling for participant-supporter interaction did not improve the conditional model fit when predicting the SF-P (χ^2^_6_=6.43, *P*=.38). Nonetheless, the results of the models controlling for participant-supporter interaction still presented a significant effect of the *tasks and goals* subscale on the estimated SF-*P* value at the end of follow-up (*γ*_02_=3.09, SE=1.30, 95% CI 0.58-5.61, t_139_=2.38, *P*=.02).

## Discussion

Responding to a general call for further studies clarifying the role of alliance in online interventions [1,9], particularly with psychometrically sound instruments [29], the aim of this study was to analyze the alliance-outcome association in a guided internet intervention for participants with moderate depression, using an adapted version of the WAI to measure the alliance in this type of approach. The results of the model showed significant effects of tasks and goals on the estimated scores of the PHQ-9 and SF-P at the end of the 9-month follow-up. These results suggest that when participants report a greater agreement with the therapeutic activities and goals proposed by the internet intervention at posttreatment, their residual depressive symptoms are lower and psychological well-being is higher at the end of follow-up. These findings are in line with the results of several studies showing the overall importance of an alliance for internet interventions [1] and the specific relevance of the alliance with an online program for treatment outcomes [18,25]. However, different from previous studies that analyzed overall alliance with internet interventions, according to suggestions from several authors [9,27,28], in this study, alliance was measured disaggregating the bond with trained supporters and the agreement with internet interventions regarding tasks and goals. Furthermore, in this study, the effects of the alliance were associated with residual depressive symptoms and patient well-being 9 months after treatment termination. However, the *tasks and goals* subscale did not exhibit a significant effect on the rate of change during the follow-up period.

Additionally, the results of this study did not show significant effects of the *bond* subscale on PHQ-9 or SF-P-estimated values at the end of follow-up or the rate of change during the follow-up period. These findings are in line with theories suggesting that in internet interventions, the agreement of tasks and goals with the intervention might be more relevant than the bond with trained supporters [9,27].

In conclusion, the results of this study point out the importance of attuning internet interventions with patients’ expectations and preferences in order to enhance their agreement with the tasks and goals of the treatment. Being responsive to patients’ needs has been presented as a fundamental process of change in face-to-face psychotherapy [44-46].

The results of this study further support this notion in internet interventions. Some online programs, such as Deprexis, use patient feedback to select specific content presented to patients [35]. However, this treatment personalization is only within modules to treat depression. Considering the high rates of comorbidities in patients with depression [47], one extension that could enhance the intervention is incorporation of modules that address other relevant symptomatologies in these patients (eg, anxiety) [27]. In the last years, there were several developments in this direction, tailoring internet interventions to the symptom profile of the patient and offering individually prescribed treatment modules accordingly [27,48-50]. The inclusion of these personalization strategies to account for possible and very likely comorbidities beyond depressive symptoms might enhance participant agreement with the tasks and goals of the program, because the intervention would further include meaningful activities to address relevant problems for the patients. Indeed, in a study comparing tailored and standardized disorder-specific internet interventions, participants who received the tailored condition rated the agreement of the tasks and goals with the program substantially and significantly higher (*P*<.001) as compared with those who received the standardized condition [27]. Future studies might need to further explore evidence-based responsiveness and treatment goals according to patient markers and analyze their associations with internet intervention processes, such as agreement on the tasks and goals of the intervention, and with acute as well as long-term outcomes.

Several limitations characterize this study. For instance, with only one assessment point for the alliance, differential effects of the general level of the alliance during treatment (ie, between-patient effects or trait-like components of alliance) and effects of the modifications of the alliance during the intervention (ie, within-patient effects or state-like components of alliance) could not be established [51,52]. Furthermore, as shown by Crits Christoph et al [53], a single measure of alliance might be less reliable than an aggregation of several measurements. The reason for assessing alliance only once at posttreatment was that contact with the supporting therapist was minimal and an adequate dose of interaction between the patient and the supporting therapist was necessary for reliable evaluation. However, this argument applies mainly to the *bond* subscale but not necessarily the *tasks and goals* agreement subscale with internet interventions (the component of the alliance that showed significant effects on long-term outcomes). Future studies would benefit from the analysis of the agreement of the tasks and goals with the intervention early in treatment and the use of repeated measures that would allow for a more fine-grained and sound analysis of the association between alliance and outcome in guided internet interventions. Additionally, although we measured alliance only at posttreatment to maximize contact between the participants and supporters before the assessment, their contact during the intervention might be too limited to fully capture the potential effects of the bond with the supporter. Future studies would need to further explore the effects of the therapeutic relationship or bond with the supporter in internet interventions where there is greater participant-supporter contact. Furthermore, the analyses of the study were conducted using a subsample of the EVIDENT randomized controlled trial (ie, patients with moderate depression who received Deprexis with email support). Further studies will need to explore whether the association between task and goal agreement and long-term outcomes can be generalized to other populations with different diagnoses (eg, anxiety disorders), different levels of severity (eg, mild or severe depression), other internet interventions, and other forms of therapist support (eg, phone support).

Despite these limitations, our study presents evidence supporting the importance of the agreement of the tasks and goals with the program in guided internet interventions for long-term outcomes. The results could inform the field to improve guided internet interventions by developing responsive strategies to adapt the interventions to patients’ specific expectations and needs and thus enhance treatment outcomes.

## Appendix

Multimedia Appendix 1 Correlations between the targeted variables of the study at posttreatment.

Multimedia Appendix 2 Results of the hierarchical linear models of the conditional main effects controlling for satisfaction or participant-supporter interaction.

## Abbreviations

PHQ-9: Patient Health Questionnaire-9
SE: standard error
SF-12: Short-Form Health Survey-12
SF-P: mental health subscale of the Short-Form Health Survey-12
WAI-I: Working Alliance Inventory for Guided Internet Interventions
ZUF-8: Zurich Satisfaction Questionnaire-8

## References

[1] Flückiger C, Del Re AC, Wampold BE, Horvath AO (2018). The alliance in adult psychotherapy: A meta-analytic synthesis. Psychotherapy (Chic).

[2] Horvath AO, Del Re AC, Flückiger C, Symonds D (2011). Alliance in individual psychotherapy. Psychotherapy (Chic).

[3] Martin DJ, Garske JP, Davis MK (2000). Relation of the therapeutic alliance with outcome and other variables: a meta-analytic review. J Consult Clin Psychol.

[4] Krupnick JL, Sotsky SM, Simmens S, Moyer J, Elkin I, Watkins J, Pilkonis PA (1996). The role of the therapeutic alliance in psychotherapy and pharmacotherapy outcome: findings in the National Institute of Mental Health Treatment of Depression Collaborative Research Program. J Consult Clin Psychol.

[5] Zuroff DC, Blatt SJ (2006). The therapeutic relationship in the brief treatment of depression: contributions to clinical improvement and enhanced adaptive capacities. J Consult Clin Psychol.

[6] Andersson G, Carlbring P, Titov N, Lindefors N (2019). Internet Interventions for Adults with Anxiety and Mood Disorders: A Narrative Umbrella Review of Recent Meta-Analyses. Can J Psychiatry.

[7] Andrews G, Basu A, Cuijpers P, Craske MG, McEvoy P, English CL, Newby JM (2018). Computer therapy for the anxiety and depression disorders is effective, acceptable and practical health care: An updated meta-analysis. J Anxiety Disord.

[8] Carlbring P, Andersson G, Cuijpers P, Riper H, Hedman-Lagerlöf E (2018). Internet-based vs. face-to-face cognitive behavior therapy for psychiatric and somatic disorders: an updated systematic review and meta-analysis. Cogn Behav Ther.

[9] Berger T (2017). The therapeutic alliance in internet interventions: A narrative review and suggestions for future research. Psychother Res.

[10] Andersson G, Paxling B, Wiwe M, Vernmark K, Felix CB, Lundborg L, Furmark T, Cuijpers P, Carlbring P (2012). Therapeutic alliance in guided internet-delivered cognitive behavioural treatment of depression, generalized anxiety disorder and social anxiety disorder. Behav Res Ther.

[11] Horvath AO, Greenberg LS (1989). Development and validation of the Working Alliance Inventory. Journal of Counseling Psychology.

[12] Anderson RE, Spence SH, Donovan CL, March S, Prosser S, Kenardy J (2012). Working alliance in online cognitive behavior therapy for anxiety disorders in youth: comparison with clinic delivery and its role in predicting outcome. J Med Internet Res.

[13] Andersson E, Ljótsson B, Hedman E, Enander J, Kaldo V, Andersson G, Lindefors N, Rück C (2015). Predictors and moderators of Internet-based cognitive behavior therapy for obsessive–compulsive disorder: Results from a randomized trial. Journal of Obsessive-Compulsive and Related Disorders.

[14] Bergman Nordgren L, Carlbring P, Linna E, Andersson G (2013). Role of the working alliance on treatment outcome in tailored internet-based cognitive behavioural therapy for anxiety disorders: randomized controlled pilot trial. JMIR Res Protoc.

[15] Hedman E, Andersson E, Lekander M, Ljótsson B (2015). Predictors in Internet-delivered cognitive behavior therapy and behavioral stress management for severe health anxiety. Behav Res Ther.

[16] Jasper K, Weise C, Conrad I, Andersson G, Hiller W, Kleinstäuber M (2014). The working alliance in a randomized controlled trial comparing Internet-based self-help and face-to-face cognitive behavior therapy for chronic tinnitus. Internet Interventions.

[17] Knaevelsrud C, Maercker A (2006). Does the quality of the working alliance predict treatment outcome in online psychotherapy for traumatized patients?. J Med Internet Res.

[18] Meyer B, Bierbrodt J, Schröder J, Berger T, Beevers CG, Weiss M, Jacob G, Späth C, Andersson G, Lutz W, Hautzinger M, Löwe B, Rose M, Hohagen F, Caspar F, Greiner W, Moritz S, Klein JP (2015). Effects of an Internet intervention (Deprexis) on severe depression symptoms: Randomized controlled trial. Internet Interventions.

[19] Preschl B, Maercker A, Wagner B (2011). The working alliance in a randomized controlled trial comparing online with face-to-face cognitive-behavioral therapy for depression. BMC Psychiatry.

[20] Richards D, Timulak L, Hevey D (2013). A comparison of two online cognitive-behavioural interventions for symptoms of depression in a student population: The role of therapist responsiveness. Counselling and Psychotherapy Research.

[21] Wagner B, Brand J, Schulz W, Knaevelsrud C (2012). Online working alliance predicts treatment outcome for posttraumatic stress symptoms in Arab war-traumatized patients. Depress Anxiety.

[22] Bordin ES (1979). The generalizability of the psychoanalytic concept of the working alliance. Psychotherapy: Theory, Research & Practice.

[23] Horvath A, Bedi R, Norcross JC (2002). The Alliance. Psychotherapy Relationships That Work.

[24] Kiluk BD, Serafini K, Frankforter T, Nich C, Carroll KM (2014). Only connect: The working alliance in computer-based cognitive behavioral therapy. Behav Res Ther.

[25] Heim E, Rötger A, Lorenz N, Maercker A (2018). Working alliance with an avatar: How far can we go with internet interventions?. Internet Interv.

[26] Ormrod JA, Kennedy L, Scott J, Cavanagh K (2010). Computerised cognitive behavioural therapy in an adult mental health service: a pilot study of outcomes and alliance. Cogn Behav Ther.

[27] Berger T, Boettcher J, Caspar F (2014). Internet-based guided self-help for several anxiety disorders: a randomized controlled trial comparing a tailored with a standardized disorder-specific approach. Psychotherapy (Chic).

[28] Scherer S, Alder J, Gaab J, Berger T, Ihde K, Urech C (2016). Patient satisfaction and psychological well-being after internet-based cognitive behavioral stress management (IB-CBSM) for women with preterm labor: A randomized controlled trial. J Psychosom Res.

[29] Gómez Penedo JM, Berger T, Grosse Holtforth M, Krieger T, Schröder J, Hohagen F, Meyer B, Moritz S, Klein JP (2019). The Working Alliance Inventory for guided Internet interventions (WAI-I). J Clin Psychol.

[30] Bockting CL, Spinhoven P, Koeter MW, Wouters LF, Schene AH, Depression Evaluation Longitudinal Therapy Assessment Study Group (2006). Prediction of recurrence in recurrent depression and the influence of consecutive episodes on vulnerability for depression: a 2-year prospective study. J Clin Psychiatry.

[31] Verhoeven FE, Wardenaar KJ, Ruhé HG, Conradi HJ, de Jonge P (2018). Seeing the signs: Using the course of residual depressive symptomatology to predict patterns of relapse and recurrence of major depressive disorder. Depress Anxiety.

[32] Wojnarowski C, Firth N, Finegan M, Delgadillo J (2019). Predictors of depression relapse and recurrence after cognitive behavioural therapy: a systematic review and meta-analysis. Behav Cogn Psychother.

[33] Klein JP, Berger T, Schröder J, Späth C, Meyer B, Caspar F, Lutz W, Greiner W, Hautzinger M, Rose M, Gräfe V, Hohagen F, Andersson G, Vettorazzi E, Moritz S (2013). The EVIDENT-trial: protocol and rationale of a multicenter randomized controlled trial testing the effectiveness of an online-based psychological intervention. BMC Psychiatry.

[34] Klein JP, Berger T, Schröder J, Späth C, Meyer B, Caspar F, Lutz W, Arndt A, Greiner W, Gräfe V, Hautzinger M, Fuhr K, Rose M, Nolte S, Löwe B, Anderssoni G, Vettorazzi E, Moritz S, Hohagen F (2016). Effects of a Psychological Internet Intervention in the Treatment of Mild to Moderate Depressive Symptoms: Results of the EVIDENT Study, a Randomized Controlled Trial. Psychother Psychosom.

[35] Meyer B, Berger T, Caspar F, Beevers CG, Andersson G, Weiss M (2009). Effectiveness of a novel integrative online treatment for depression (Deprexis): randomized controlled trial. J Med Internet Res.

[36] Kroenke K, Spitzer RL, Williams JB, Löwe B (2010). The Patient Health Questionnaire Somatic, Anxiety, and Depressive Symptom Scales: a systematic review. Gen Hosp Psychiatry.

[37] Twomey C, O'Reilly G, Meyer B (2017). Effectiveness of an individually-tailored computerised CBT programme (Deprexis) for depression: A meta-analysis. Psychiatry Res.

[38] Berger T, Hämmerli K, Gubser N, Andersson G, Caspar F (2011). Internet-based treatment of depression: a randomized controlled trial comparing guided with unguided self-help. Cogn Behav Ther.

[39] Munder T, Wilmers F, Leonhart R, Linster HW, Barth J (2010). Working Alliance Inventory-Short Revised (WAI-SR): psychometric properties in outpatients and inpatients. Clin Psychol Psychother.

[40] Schmidt J, Lamprecht F, Wittmann WW (1989). [Satisfaction with inpatient management. Development of a questionnaire and initial validity studies]. Psychother Psychosom Med Psychol.

[41] Kroenke K, Spitzer RL, Williams JB (2001). The PHQ-9: validity of a brief depression severity measure. J Gen Intern Med.

[42] Ware J, Kosinski M, Keller SD (1996). A 12-Item Short-Form Health Survey: construction of scales and preliminary tests of reliability and validity. Med Care.

[43] Raudenbush S, Bryk AS (2001). Hierarchical Linear Models: Applications And Data Analysis Methods (Advanced Quantitative Techniques in The Social Sciences).

[44] Constantino M, Boswell J, Bernecker S, Castonguay L (2013). Context-responsive psychotherapy integration as a framework for a unified clinical science: Conceptual and empirical considerations. J Unified Psychother Clin Sci.

[45] Gomez Penedo JM, Constantino MJ, Coyne AE, Westra HA, Antony MM (2017). Markers for context-responsiveness: Client baseline interpersonal problems moderate the efficacy of two psychotherapies for generalized anxiety disorder. J Consult Clin Psychol.

[46] Stiles W, Honos-Webb L, Surko M (1998). Responsiveness in psychotherapy. Clin Psychol Sci Pract.

[47] Kessler RC, Zhao S, Blazer DG, Swartz M (1997). Prevalence, correlates, and course of minor depression and major depression in the National Comorbidity Survey. J Affect Disord.

[48] Andersson G, Estling F, Jakobsson E, Cuijpers P, Carlbring P (2011). Can the patient decide which modules to endorse? An open trial of tailored internet treatment of anxiety disorders. Cogn Behav Ther.

[49] Carlbring P, Maurin L, Törngren C, Linna E, Eriksson T, Sparthan E, Strååt M, Marquez von Hage C, Bergman-Nordgren L, Andersson G (2011). Individually-tailored, Internet-based treatment for anxiety disorders: A randomized controlled trial. Behav Res Ther.

[50] Johansson R, Sjöberg E, Sjögren M, Johnsson E, Carlbring P, Andersson T, Rousseau A, Andersson G (2012). Tailored vs. standardized internet-based cognitive behavior therapy for depression and comorbid symptoms: a randomized controlled trial. PLoS One.

[51] Falkenström F, Finkel S, Sandell R, Rubel JA, Holmqvist R (2017). Dynamic models of individual change in psychotherapy process research. J Consult Clin Psychol.

[52] Zilcha-Mano S (2017). Is the alliance really therapeutic? Revisiting this question in light of recent methodological advances. Am Psychol.

[53] Crits-Christoph P, Gibbons MB, Hamilton J, Ring-Kurtz S, Gallop R (2011). The dependability of alliance assessments: the alliance-outcome correlation is larger than you might think. J Consult Clin Psychol.

