# Supplementary material for: The Association of Therapeutic Alliance With Long-Term Outcome in a Guided Internet Intervention for Depression: Secondary Analysis From a Randomized Control Trial
Source: J Med Internet Res. 2020 Mar 24;22(3):e15824. doi: 10.2196/15824 (PMC7139432; doi:10.2196/15824)
Supplement: Multimedia Appendix 1 [file jmir_v22i3e15824_app1.docx]

**Multimedia Appendix**

**Multimedia Appendix 1**

| Multimedia Appendix 1. Correlations between the targeted variables of the study at post-treatment. | | | | |
| --- | --- | --- | --- | --- |
| *Variables* | 1 | 2 | 3 | 4 |
| 1 WAI-I^a^ T&G^b^ | - |  |  |  |
| 2 WAI-I Bond | .61^f^ | - |  |  |
| 3 ZUF-8^c^ | .82^f^ | .54^f^ | - |  |
| 4 PHQ-9^d^ | -.44^f^ | -.25^f^ | .33^f^ | - |
| 5 SF-P^e^ | .36^f^ | .20^f^ | -.43^f^ | -.70^f^ |
| *Note.* ^a^WAI-I = Working Alliance Inventory for Internet Interventions; ^b^T&G = Task & Goal subscale; ^c^ZUF-8 = Patient satisfaction questionnaire; ^d^PHQ = Patient Health Questionnaire; ^e^SF-P = Mental health well-being subscale of the Short-Form Health Survey −12; ^f^*P* < .001. | | | | |
