# Supplementary material for: The Association of Therapeutic Alliance With Long-Term Outcome in a Guided Internet Intervention for Depression: Secondary Analysis From a Randomized Control Trial
Source: J Med Internet Res. 2020 Mar 24;22(3):e15824. doi: 10.2196/15824 (PMC7139432; doi:10.2196/15824)
Supplement: Multimedia Appendix 2 [file jmir_v22i3e15824_app2.docx]

**Multimedia Appendix**

**Multimedia Appendix 2**

| Multimedia Appendix 2. Results of the conditional main effects’ hierarchical linear models controlling for satisfaction or participant-supporter interaction | | | | | | | |
| --- | --- | --- | --- | --- | --- | --- | --- |
|  |  | Estimated score at the end of follow-up (*β_0j_*) | |  | Change during follow-up (*β_1j_*) | |  |
| Fixed Effects |  | γ | *SE* |  | γ | *SE* |  |
| **PHQ as outcome** |  |  |  |  |  |  |  |
| *Main effects of alliance*  *controlling for satisfaction* |  |  |  |  |  |  |  |
| Intercept |  | 6.82^d^ | 0.28 |  | -0.49^f^ | 0.27 |  |
| WAI-I T&G |  | -1.78^e^ | 0.58 |  | -0.70 | 0.55 |  |
| WAI-I Bond |  | 0.56^f^ | 0.32 |  | 0.46 | 0.31 |  |
| ZUF-8 Total |  | 0.09 | 0.85 |  | 2.00^e^ | 0.81 |  |
| *Main effects of alliance*  *controlling for participant-supporter interaction* |  |  |  |  |  |  |  |
| Intercept |  | 7.23^d^ | 0.31 |  | -0.55 | 0.34 |  |
| WAI-I T&G |  | -1.97^e^ | 0.44 |  | 0.06 | 0.47 |  |
| WAI-I Bond |  | 0.67^f^ | 0.35 |  | 0.75^e^ | 0.38 |  |
| Messages-P^a^ |  | 0.03 | 0.15 |  | 0.04 | 0.16 |  |
| Messages-S^b^ |  | -0.02 | 0.18 |  | -0.01 | 0.19 |  |
| Messages read^c^ |  | 0.04 | 0.11 |  | 0.02 | 0.12 |  |
| **SF-P as outcome** |  |  |  |  |  |  |  |
| *Main effects of alliance*  *controlling for satisfaction* |  |  |  |  |  |  |  |
| Intercept |  | 40.19^d^ | 0.79 |  | 1.39^f^ | 0.76 |  |
| WAI-I T&G |  | 2.54 | 1.64 |  | 1.18 | 1.58 |  |
| WAI-I Bond |  | -0.78 | 0.92 |  | -0.35 | 0.89 |  |
| ZUF-8 Total |  | -1.11 | 2.37 |  | -0.18 | 2.30 |  |
| *Main effects of alliance*  *controlling for participant-supporter interaction* |  |  |  |  |  |  |  |
| Intercept |  | 38.72^d^ | 0.92 |  | 1.73^f^ | 0.94 |  |
| WAI-I T&G |  | 3.09^e^ | 1.30 |  | -0.93 | 1.33 |  |
| WAI-I Bond |  | -0.69 | 1.04 |  | -1.07 | 1.07 |  |
| Messages-P^a^ |  | -0.21 | 0.46 |  | -0.69 | 0.47 |  |
| Messages-S^b^ |  | 0.28 | 0.52 |  | 0.04 | 0.54 |  |
| Messages read^c^ |  | -0.28 | 0.32 |  | 0.30 | 0.34 |  |
| *Note.* See references in Appendix 1. ^a^Messages sent by the participant; ^b^Messages sent by the supporter, ^c^Messages read by the participant, ^d^*P* < .001, ^e^*P* < .01, ^f^*P* < .10. | | | | | | | |
